# The functions and roles of sestrins in regulating human diseases

**DOI:** 10.1186/s11658-021-00302-8

**Published:** 2022-01-03

**Authors:** Yitong Chen, Tingben Huang, Zhou Yu, Qiong Yu, Ying Wang, Ji’an Hu, Jiejun Shi, Guoli Yang

**Affiliations:** 1 Department of Orthodontics, Stomatology Hospital, School of Stomatology, Zhejiang University School of Medicine, Clinical Research Center for Oral Diseases of Zhejiang Province, Key Laboratory of Oral Biomedical Research of Zhejiang Province, Cancer Center of Zhejiang University, Hangzhou, 310006 Zhejiang China; 2 Department of Implantology, Stomatology Hospital, School of Stomatology, Zhejiang University School of Medicine, Clinical Research Center for Oral Diseases of Zhejiang Province, Key Laboratory of Oral Biomedical Research of Zhejiang Province, Cancer Center of Zhejiang University, Hangzhou, 310006 Zhejiang China; 3 Department of Oral Medicine, Stomatology Hospital, School of Stomatology, Zhejiang University School of Medicine, Clinical Research Center for Oral Diseases of Zhejiang Province, Key Laboratory of Oral Biomedical Research of Zhejiang Province, Cancer Center of Zhejiang University, Hangzhou, 310006 Zhejiang China; 4 Department of Oral Pathology, Stomatology Hospital, School of Stomatology, Zhejiang University School of Medicine, Clinical Research Center for Oral Diseases of Zhejiang Province, Key Laboratory of Oral Biomedical Research of Zhejiang Province, Cancer Center of Zhejiang University, Hangzhou, 310006 Zhejiang China

**Keywords:** Sestrins, Biological functions, Human diseases, Musculoskeletal system disease, Biomarker, Therapeutic target

## Abstract

Sestrins (Sesns), highly conserved stress-inducible metabolic proteins, are known to protect organisms against various noxious stimuli including DNA damage, oxidative stress, starvation, endoplasmic reticulum (ER) stress, and hypoxia. Sesns regulate metabolism mainly through activation of the key energy sensor AMP-dependent protein kinase (AMPK) and inhibition of mammalian target of rapamycin complex 1 (mTORC1). Sesns also play pivotal roles in autophagy activation and apoptosis inhibition in normal cells, while conversely promoting apoptosis in cancer cells. The functions of Sesns in diseases such as metabolic disorders, neurodegenerative diseases, cardiovascular diseases, and cancer have been broadly investigated in the past decades. However, there is a limited number of reviews that have summarized the functions of Sesns in the pathophysiological processes of human diseases, especially musculoskeletal system diseases. One aim of this review is to discuss the biological functions of Sesns in the pathophysiological process and phenotype of diseases. More significantly, we include some new evidence about the musculoskeletal system. Another purpose is to explore whether Sesns could be potential biomarkers or targets in the future diagnostic and therapeutic process.

## Introduction

Sesns comprise an evolutionarily conserved family of proteins universally found in animals. They are encoded by genes highly expressed in cells exposed to a variety of stresses, including oxidative stress, DNA damage, hypoxia, and starvation [1–3]. Vertebrates express three distinct Sesns (SESN1, SESN2, and SESN3) [1]. SESN1 is a member of the growth arrest and DNA damage-inducible gene (GADD) family [2, 4]. It is ubiquitously expressed in human tissues, mostly in skeletal muscle, heart, liver, and brain [5]. SESN2, known as hypoxia-inducible gene 95, is upregulated in cells under hypoxic conditions as well as oxidative stress, DNA damage, endoplasmic reticulum stressors, starvation, and high-fat diet [6–8]. It has also been identified as a key leucine sensor for the mTORC1 pathway in mammalian cells [9, 10]. SESN2 is highly expressed in kidney, lungs, leukocytes, liver, gastrointestinal tract, and brain [5]. SESN1 and SESN2 are regulated by the tumor-suppressor protein p53, while SESN3, the least reported one of the family, is majorly activated by FoxO transcription factors [11, 12]. SESN3 is highly expressed in brain, kidney, colon, small intestine, liver, and skeletal muscle [13, 14]. In normal physiology and organ homeostasis, Sesns control important cellular processes, including tissue growth, antioxidant response, metabolic homeostasis, nutrient sensing, autophagy, protein synthesis, and age-related pathologies. Different pathways are involved in the mechanisms of these processes, such as the AMPK/mTORC1 pathway, GATOR-Rags pathway, Keap1-Nrf2 pathway, and mTORC2-AKT pathway [14]. The structures, regulators, and functions of human Sesns are summarized in Table 1.

**Table 1:** Structure and functions of human sestrins

Sestrins	Transcript variant	Crystal structure	Regulators	Functions	References
hSesn1	3 (~ 48,55,68 kDa)	Unknown (composed mostly of α-helical regions)	p53, FoXO	① Inhibition of ROS ② Nutrition sensing (amino acid, glucose, leucine) ③ Inhibition of mTORC1 ④ Induction of autophagy	[48, 104, 163, 194]
hSesn2	1 (~ 60 kDa)	Two-fold pseudosymmetry with 3 subdomains	p53, Nrf2, ATF4, C/EBPβ, JNK/c-Jun, AP-1, HIF1	① Inhibition of ROS, DNA damage, and ER stress ② Nutrition sensing (amino acid, glucose, leucine) ③ Inhibition of cell growth and mTORC1 ④ Induction of autophagy ⑤ Maintaining homeostasis of glucose, insulin, fatty acid, and triglyceride	[6, 9, 10, 22, 42, 48, 57, 86, 136, 162, 194]
hSesn3	2 (44,53 kDa)	Unknown	AP-1, FoxO1, FoxO3	① Inhibition of ROS ② Nutrition sensing (amino acid, glucose, and leucine) ③ Regulation of mTORC1/mTORC2/PKB ④ Induction of autophagy ⑤ Maintaining homeostasis of glucose, insulin	[22, 39, 194]

Sesn, Sestrin; Nrf2, nuclear factor erythroid 2-related factor 2; AP-1, activator protein 1; ATF4, activating transcription factor 4; C/EBPβ, CCAAT/enhancer-binding protein beta; JNK, c-Jun N-terminal kinase; HIF1, hypoxia-inducible factor 1; FoxO, forkhead box protein O; ROS, reactive oxygen species; mTORC1, mechanistic target of rapamycin complex 1; mTORC2, mechanistic target of rapamycin complex 2; PKB, protein kinase B, also known as Akt; AMPK, AMP-activated protein kinase

Identified as substantial anti-aging genes and regulators of reactive oxygen species (ROS) and mammalian targets of rapamycin complex 1 (mTORC1) [15], Sesns are associated with many age-related diseases, including cardiovascular diseases (CVDs), neurodegenerative diseases, chronic respiratory diseases, intervertebral disc degeneration (IDD), sarcopenia, etc. [2, 3, 16, 17]. As regulators of cellular homeostasis, Sesns are also connected with diseases such as diabetes, obesity, obstructive sleep apnea (OSA), neuropathic pain, epilepsy, and osteoarthritis. [16, 18–20].

In this review, we summarize the latest advances regarding the biological functions of human Sesns. In addition, the roles of Sesns in the pathophysiology of different human body systems and organs are discussed. Furthermore, we introduce the evidence of Sesns as potential biomarkers and therapeutic targets for various diseases. Nonetheless, most studies on Sesns are still in the experimental stages; thus, there is a long way to go before Sesns can be applied in clinical diagnosis and treatment. This article provides a comprehensive review on the roles of Sesns in the pathogenesis, diagnosis, and treatment of human diseases, and offers an outlook on future directions in Sesns research.

## Biological functions of Sesns in human pathophysiological processes

Numerous studies have demonstrated that Sesns protect organisms against various pathologies, such as aging, metabolic homeostasis, lipid accumulation, and insulin resistance [21]. Sesns perform various biological functions by responding to different internal and external environmental stressors, including oxidative stress, genotoxic stress, hypernutrition, starvation, hypoxia, ER stress, etc. [2, 3, 21–23]. Here, we summarize the functions of Sesns under different unfavorable conditions (Table 2).

**Table 2:** Biological functions of sestrins in pathophysiological processes

Conditions	Upstream pathways	Sestrins	Downstream pathways	Functions	References
Oxidative stress	p53, Nrf2/ARE, FoxO1, FoxO3, JNK/AP-1, PERK-C/EBPβ, NMDA receptor	Sesn1/2/3	AMPK/mTORC1, Nrf2, peroxiredoxin AhpC, Nox4, PDGFRβ, p38 MAPK, dopamine D2 receptor, and FoxO1	Increased expression of antioxidant enzymes (trigger antioxidant response)	[1, 14, 35, 36, 38–44]
Genotoxic stress	p53, FoxO3	Sesn1/2/3	AMPK/mTOR, AMPK/TOR, p-Beclin1-Parkin, JNK, PGC-1α	DNA repair	[8, 48]
Carcinogenesis	p53	Sesn2	mTORC2/Akt	Oncogenesis	[53]
Hypernutrition	Glucose, insulin, fatty acid, and triglyceride	Sesn1/2/3	AMPK, mTORC1-S6K, mTORC2/Akt	Maintain lipid and glucose homeostasis	[1, 13, 22, 57, 58]
Nutrient starvation	ATF4, Nrf2, JNK/ c-Jun, FoxO1, FoxO3, PGC-1α	Sesn1/2	mTORC1	Inhibition of necrosis and apoptosis in cells, represses majority of protein translation, growth regulation, autophagy induction, regulate cellular energy homeostasis	[35, 37]
Hypoxia	p53, HIF-1, PI3K/Akt	Sesn2	VEGF, AMPK-PHD	Reduce hypoxic damage	[6, 8, 54, 68]
ER stress	PERK, PERK-C/EBPβ, ATF4/Nrf2, IRE1/XBP1, ATF6	Sesn2	AMPK/mTORC1, c/EBP homologous protein, p38, JNK, UPR, PERK-ATF4-CHOP	Maintains autophagy homeostasis and prevents apoptosis	[71–79]
Autophagy dysregulation	AMPK/mTORC1, p53	Sesn2	AMPK/mTOR, PI3K/Akt/mTOR, AMPKα, mTORC1-ULK1-S6, autophagy protein p62/SQSTM1,	Autophagy induction	[1, 15, 82, 83]
Mitochondrial dysfunction	ATF4, RBX1, p53	Sesn2	AMPK/mTOR, AMPK/TOR, p-Beclin1-Parkin, JNK, PGC-1α	Mitophagy induction	[85–87]
Immune dysregulation	AMPK/mTORC1	Sesn2	NLRP3 inflammasome; Erk-JNK-p38 MAPK; AP-1, ULK1, SQSTM1, AMPK/ERs	Hyper-inflammation; T-cell senescence; anti-inflammation	[62, 75, 79, 85, 89–91, 94–97]

Sesn, sestrin; Nrf2, nuclear factor erythroid 2-related factor 2; ARE, antioxidant responsive element; FoxO, forkhead box protein O; JNK, c-Jun N-terminal kinase; AP-1, activator protein 1; PERK, protein kinase RNA-like endoplasmic reticulum kinase; C/EBPβ, CCAAT/enhancer-binding protein beta; NMDA, N-methyl-d-aspartate; AMPK, AMP-activated protein kinase; mTORC1, mechanistic target of rapamycin complex 1; Nox4, NADPH oxidase 4; PDGFRβ, platelet-derived growth factor receptor beta; MAPK, mitogen-activated protein kinase; Beclin1, mammalian homolog of yeast ATG6; PGC-1α, peroxisome proliferator-activated receptor-gamma coactivator alpha; mTORC2, mechanistic target of rapamycin complex 2; S6K, ribosomal protein S6 kinase; HIF1, hypoxia-inducible factor 1; PI3K, phosphoinositide 3-kinase; PHD, prolyl hydroxylase; ATF4, activating transcription factor 4; IRE1, inositol-requiring enzyme 1; XBP1, X-box binding protein 1; ATF6, activating transcription factor 6; UPR, unfolded protein response; PERK, PKR-like ER kinase; CHOP, C/EBP homologous protein; ULK1, unc-51 like autophagy activating kinase 1; SQSTM1, sequestosome 1; RBX1, ring-box 1; NLRP3, Nod-like receptor family pyrin domain containing 3; Erk, extracellular signal-regulated kinase; ERs, estrogen receptors

### Oxidative stress

Oxidative stress is a phenomenon caused by the accumulation of excess reactive nitrogen species (RNS), ROS, and other reactive metabolic intermediates, which overwhelms the antioxidant system in living organisms [1, 3, 14]. Oxidative stress can cause damage to DNA, RNA, and other molecules such as proteins and lipids, contributing to aging, cell apoptosis, cardiovascular diseases, chronic kidney disease, neurodegenerative diseases, metabolic syndrome, etc. [3, 24–28]. Sesns are reported to be induced by oxidative stress in pathological conditions such as heart failure, colorectal diseases, atrial fibrillation, diabetes, cancer, chronic obstructive pulmonary disease, Alzheimer’s disease (AD), and Parkinson’s disease (PD) [14, 29–34]. Different mechanisms are reported to be involved in Sesns antioxidant reaction. Based on current evidence, SESN1 is induced by oxidative stress in a p53-dependent manner. SESN2 is activated not only in a p53-dependent manner, but through the NMDA receptor pathway, Nrf2 pathway, and JNK-AP-1 signaling axis as well [1, 35, 36]. The *SESN2* gene is activated by mitochondrial specific ROS and dictates JNK specific inactivation of the apoptotic pathway [37]. SESN3 is stimulated by oxidative damage via activation of FoxO transcription factors [38, 39]. Although the mechanism of Sesns’ antioxidant function is still unclear, several proteins or pathways may contribute to this process. They include the regeneration of peroxiredoxin AhpC, mTORC1&Nox4 [13, 40, 41], the Keap1-Nrf2 pathway [3, 36], inhibition of uncoupling protein 1 expression by suppressing p38 MAPK [42], the dopamine D2 receptor [43], and the Akt/FoxO1 axis [44].

### Genotoxic stress

Genotoxic stress is suggested to boost aging and activate DNA damage through mutations or genomic instability [45]. Also, it is a common challenge for cells exposed to toxic agents, including ultraviolet rays, chemotherapeutics agents, ionizing radiation, and overproduction of highly reactive molecules such as ROS, lipid peroxidation products, and DNA-alkylating agents [3, 46, 47]. SESN1 and SESN2 can both respond to genotoxic stress in a p53-dependent manner [8]. The ability of SESN1/2 to protect cells against DNA damage may be attributed to their redox activity and their redox-independent ability of inhibiting mTOR signaling [48]. Furthermore, Sesn2 can save energy from protein translation and membrane synthesis for DNA repair by activation of AMPK and inhibition of mTOR signaling [14].

### Carcinogenesis

Carcinogenesis or tumorigenesis may be initiated and promoted by an imbalance between cell-intrinsic responses of target cells and changes in the tumor microenvironment caused by genotoxic stress [49]. Considering the ability of Sesns in inhibiting genotoxic damage and the oncogenic mTOR pathway, the role of Sesns in carcinogenesis is expected [48, 50]. Previous studies on colon cancer, lung carcinoma, and lung adenocarcinoma have proved the tumor-suppressive functions of Sesns [2, 51, 52]. Surprisingly, Sesns are also vital in maintaining the viability of cancers under specific conditions. These cancers include squamous cell carcinoma (SCC), melanoma and hepatocellular carcinoma [37, 53]. The oncogenic function of Sesns may be ascribed to their protection against energetic stress via Akt and mTOR signaling [54]. Further studies are needed to elucidate the dual role of Sesns in different neoplastic diseases for potential anti-tumor therapeutic approaches.

### Hypernutrition

Hypernutrition promotes the development of obesity and metabolic syndromes such as type 2 diabetes, insulin resistance, and elevated blood glucose levels [1, 55]. Sesns have been reported to be induced in organs such as the muscle, adipose tissue, and liver in animal models of type 2 diabetes and obesity [22, 56]. A previous study reported that SESN2-deficient obesity mouse developed glucose intolerance, insulin resistance, and hepatosteatosis, all of which were augmented by mTORC1-S6K activation in response to nutritional abundance [1, 57]. Tao et al. demonstrated that SESN3 protected high-fat-fed mice against insulin resistance through the mTORC2/Akt pathway [13]. Therefore, evidence suggests that Sesns are essential in maintaining metabolic homeostasis and protecting against hypernutrition [13, 22, 58–61].

### Nutrient starvation

SESN2 is the major Sesns family member that is activated under nutrient starvation [54]. Upon energy deprivation, SESN2 protects against cell apoptosis, and regulates protein synthesis and autophagy via the AMPK/mTORC1 pathway [62–64]. This proves that SESN2 is a crucial nutrient sensor that modulates energy homeostasis. Due to these functions, activation of Sesns may enhance the survival of tumor cells under the condition of limited nutrition. Different mechanisms are involve in Sesns induction by deficiency of different nutrients. Under glucose scarcity, SESN2 elevation may depend on PGC-1α activation [37]. Under serum deprivation, the c-Jun N-terminal kinase (JNK) pathway activation and its downstream factor c-Jun phosphorylation may activate the expression of Sesns [35].

### Hypoxia

Hypoxia is one of the most severe metabolic insults, which is associated with a variety of pathological conditions, such as pulmonary arterial hypertension, arrhythmia, hypoxic-ischemic encephalopathy (HIE), myocardial ischemia injury and cancer [65, 66]. SESN1 and SESN2 can be induced by hypoxia in many human cancer cell lines [1]. The pathways vary among the isoforms. SESN1 is activated strictly in a p53-dependent manner [54], while SESN2 could be activated by hypoxia through the HIF-1-dependent pathway [67] and HIF-1-independent pathway [8]. The PI3K/Akt pathway may also be involved in the Sesn2 transcriptional process [54]. Sesns are found to protect against several hypoxia related pathological conditions. Harmful chemicals such as 2-deoxyglucose and metformin (an inhibitor of mitochondrial respiration) stimulate the expression of SESN2 [54]. In hypoxic-ischemic mouse models, SESN2 was found to inhibit VEGF production and attenuate the blood–brain barrier permeability to reduce brain damage [68]. A previous study with colorectal cancer cells and mouse xenograft models suggested that SESN2 inhibited tumorigenesis by promoting the degradation of HIF-1α via AMPK-PHD regulation [6].

### ER stress

ER stress occurs when misfolded proteins accumulate due to pathological conditions in normal aging and a variety of degenerative diseases, such as cancer, obesity, PD, AD, IDD, and sarcopenia [69, 70]. ER stress causes tissue damage by impairing a series of molecular and biochemical processes, including protein folding and protein transportation [7].

Studies have shown that ER stress induces SESN2 expression through the PERK and IRE1/XBP1 transduction pathways [3]. Ding et al. demonstrated that glucose starvation activated SESN2 via ATF4 and Nrf2 activation [71]. In the absence of SESN2, cells are highly susceptible to ER-related pathologies, including mitochondrial dysfunction, lipid accumulation, protein aggregate formation, and apoptosis [72–74]. SESN2 acts as a crucial regulator in ER stress-related atherosclerosis [75], liver injury [76, 77], spinal cord injury [78], and sepsis-related dendritic cell apoptosis [79]. The AMPK/mTORC1 pathway, CCAAT-enhancer-binding protein homologous protein, phosphorylation of both p38 and JNK, and sestrin-mediated unfolded protein response contribute to the protective mechanisms of SESN2 against ER stress-associated diseases [72, 75–77].

### Autophagy dysregulation

Autophagy refers to cellular mechanisms by which cells break down and recycle damaged or toxic cellular structures to maintain organelle function and cell homeostasis [80]. Autophagy impairment comes from accumulation of protein aggregates, damaged mitochondria, and ROS. This deficiency can result in diverse neurodegenerative diseases, such as Parkinson’s, Alzheimer’s, and Huntington’s diseases [81]. Sesns promote autophagy by activating AMPK and inhibiting mTORC1, thus attenuating neurodegenerative diseases [15, 22, 82]. In addition, the induction of autophagy by p53-SESN2 enhances anticancer processes in various human carcinoma cells [83].

Mitophagy is a specific form of autophagy that is vital in ensuring the functionality and integrity of the mitochondrial network. Dysregulation of mitophagy results in mitochondrial ROS accumulation and is crucial in diverse degenerative pathologies such as PD, AD, Leber’s hereditary optic neuropathy, inflammation, sepsis aging, and cancer [5, 84]. As a positive regulator of Parkin-mediated autophagy, SESN2 is essential in mitochondrial homeostasis [5]. SESN2 can regulate mitophagy by enhancing the targeting of impaired mitochondria for lysosomal degradation and via regulation of Parkin E3 ligase migration to damaged mitochondrial surface [85, 86]. It is demonstrated that the p53-SESN2 axis provides a protective mechanism against acute kidney injury by regulating autophagy and mitophagy in renal tubules [87]. Kumar et al. found that SESN2 could promote cell death under long-term mitochondrial damage rather than regulating the mitophagy upon normal mitochondrial stress [88].

### Immune dysregulation

Immune dysregulation is associated with a variety of diseases, including infections and malignancy [89]. Recently, the involvement of SESN2 in immune cells, including macrophages, monocytes, T cells, NK cells, and B cells, has been studied [54, 90]. SESN2 protected macrophages from apoptosis and alleviated the excessive inflammatory response of macrophages in diseases such as myocardial infarction [91–93]. Monocytes can also be regulated by Sesn2 to reduce the damage caused by LPS-induced inflammation, atherosclerosis, high-glucose status, high-fat condition, and sepsis [76, 84, 94]. Mechanically, *SESN2* knockdown significantly increases the secretion of pro-inflammatory cytokines, regulates monocyte polarization, and increases monocyte recruitment to the vascular endothelial cells by downregulating AMPK signaling and the ER stress pathway. Additionally, SESN2 maintains immunological homeostasis by activating mitophagy in monocytes to restrain the NLRP3 inflammasome hyperactivation [85].

However, the effects of Sesns in T cells may oppose their functions in other cells [54]. Sesns expression and Sesn–MAPK activation immune-inhibition complex levels were higher in T cells from older humans and mice [95]. Inhibition of SESN1/2/3 in senescent T cells enhanced cell proliferation, telomerase activity, and IL-2 synthesis viability, demonstrating an anti-aging effect [21, 95]. A recent study in the acute colitis mouse model suggested that SESN3 might be vital in generating pathogenic Th1 and Th17 cells mediated by macrophage in inflammatory bowel diseases [96].

Also, Sesns could induce the reprogramming of non-proliferative senescent-like CD8(+) T cells to acquire a natural killer function, which may be vital to surveilling and eliminating senescent cells during aging [89]. In ovarian cancer cells, SESN2 and SESN3 restrained NK cell-mediated cytotoxic activity through the AMPK and mTORC1 signaling [97]. Experimental results in mouse B cells indicate that SESN2 may be a therapeutic target in IgE-mediated allergic diseases since SESN2-AMPK signaling selectively promotes IgE class switching and IgE production [90]. In dendritic cells, SESN2 also exerts a protective effect against sepsis by inhibiting apoptotic ER stress signaling [79].

## The roles of Sesns in human diseases

**Fig. 1:**
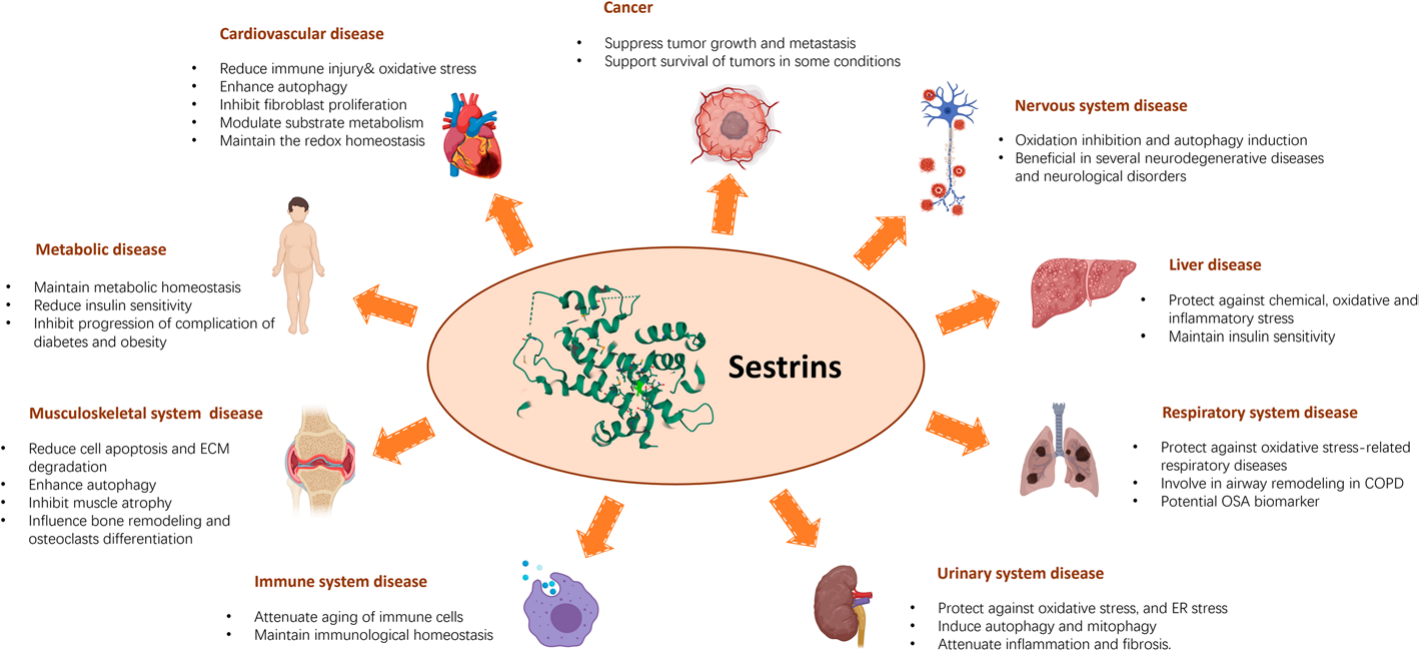
Roles of Sesns in human diseases. Sesns are proved to regulate cell metabolism and cellular homeostasis with their biological functions of protecting against various kinds of environmental stressors and regulating the AMPK/mTORC1 pathway. The protective and harmful effects of Sesns in various age-related diseases, metabolic disorders, and cancers are shown in this figure. COPD, chronic obstructive pulmonary disease; ER, endoplasmic reticulum; ECM, extracellular matrix

Sesns protect against various environmental stressors and regulate the AMPK/mTORC1 pathway. Moreover, Sesns regulate cell metabolism and cellular homeostasis in both normal and diseased states [70]. The protective effects of Sesns in all sorts of human diseases have significantly attracted researchers (Fig. 1).

### Cardiovascular diseases

CVDs are the main cause of death worldwide [98]. Studies have shown that Sesns play important protective roles in various CVDs, including atherosclerosis (AS), acute myocardial infarction (AMI), heart failure, hypertension, myocardial hypertrophy, atrial fibrillation, and myocardial fibrosis [3, 99, 100]. The role of Sesns in CVDs is associated with their versatile functions in cardiology. Their functions include reducing ROS level, alleviating inflammation, and attenuating aging. Sesns could also enhance autophagy, inhibit fibroblast proliferation, modulate substrate metabolism, and maintain the redox homeostasis [3, 98, 99].

CVDs such as heart failure, atrial fibrillation, hypertension, and AS are strongly associated with excessive ROS [3]. Sesns can reduce production of ROS via mTORC1-independent mechanisms and protect cells against ROS accumulation by promoting the cyclic absorption of over-oxidized peroxidase [42, 43]. This signifies the exciting potential for therapeutic and diagnostic applications in CVDs [92]. In patients with coronary heart disease (CAD), aortic dissection, and chronic heart failure (CHF), the level of SESN2 is elevated and appears to be related to the severity of the disease [101–103].

The macrophage-mediated inflammatory response also has important roles in CVDs such as AS, AMI and heart failure [3, 33]. M1 macrophages promote secretion of matrix metalloproteinases and pro-inflammatory factors to promote the development of CVDs, while M2 macrophages tend to secrete anti-inflammatory factors [8, 13]. Sesns affect the level of inflammatory factors and regulate the M1/M2 macrophage balance via the AMPK-mTOR pathway and eventually result in an anti-inflammatory response [3, 12, 24, 104].

Myocardial ischemia and reperfusion (I/R) injury may lead to cardiac arrhythmia and heart failure [105]. Sesns may protect the cardiovascular system against I/R injury by attenuating ROS accumulation and enhancing autophagy [106]. Additionally, Sesns could prevent age-related intolerance to ischemic heart disease by LKB1-mediated AMPK activation and substrate metabolism modulation [107, 108].

### Metabolic diseases

Metabolic disorders, such as obesity-associated CVDs, diabetes, and non-alcoholic fatty liver disease, are marked by the regulation of AMPK and mTOR [2]. Sesns have been demonstrated to play a critical role in metabolic control and glucose homeostasis by regulating AMPK/mTORC1 [109]. In contrast, the effects of SESN3 on insulin sensitivity and glucose metabolism is probably associated with mTORC2-Akt signaling with little involvement of AMPK [14]. Different Sesns isoforms have different responses to metabolic disorders. SESN2 accumulated in the muscle, liver, and adipose tissues in a mouse model of type 2 diabetes and obesity [22], whereas SESN1 decreased in the skeletal muscle and SESN3 decreased in the liver and adipose tissue in patients with high-fat diet and diabetics [29]. Lack of SESN2 increased the progression of diabetes, obesity-induced insulin resistance, and the severity of hepatosteatosis caused by obesity [22]. Moreover, a recent report showed that exercise could induce SESN2 and increase insulin sensitivity through autophagy [110].

### Nervous system diseases

Various neurodegenerative diseases and neurological disorders are related to excessive oxidative stress with compromised antioxidant capacity and accumulation of misfolded proteins [111, 112]. Due to their biological functions in anti-oxidation and autophagy promotion, protective roles of Sesns are gradually appreciated in neurodegenerative diseases and neurological disorders [19, 113–116]. The former diseases include AD, PD, Huntington’s disease (HD), and amyotrophic lateral sclerosis (ALS). The latter refer to seizures, neuropathic pain, ischemic stroke, and neonatal hypoxic-ischemic encephalopathy. The evidence of SESN1 and SESN3 in regulating the nervous system is relatively scarce since most studies focused on SESN2. This calls for further investigation into the potentially unique functions of these two proteins [113].

### Liver diseases

The liver is a metabolically active organ that is susceptible to oxidative damage. Since Sesns are regarded as key inhibitors of oxidative stress, the roles of Sesns in liver diseases have been widely investigated. Sesns are associated with various liver diseases, including hepatocyte injury, hepatitis, nonalcoholic fatty liver disease (NAFLD), and liver cancers such as hepatocellular carcinoma (HCC) [117]. The hepatoprotective effect of SESN2 may be due to its regulation upon the Nrf2/Keap1 pathway to reduce the liver’s susceptibility to oxidative damage [118]. Inhibition of mitochondrial dysfunction and remittance of ER stress-associated liver damage may also explain this effect [118]. The action of Sesns against hepatic metabolic stress, liver infectious disease and HCC is discussed in other sections. Accumulated reports indicate that Sesns may be promising targets in liver disease; however, the exact mechanisms of action of Sesns against liver diseases are still unclear [118].

### Respiratory system diseases

Recent studies have shown that Sesns are involved in many oxidative stress-related respiratory diseases, including chronic obstructive pulmonary disease (COPD), asthma, acute respiratory distress syndrome (ARDS), OSA, and CS-induced emphysema [119–121].

SESN2 is upregulated in the lungs of COPD patients and mutational inactivation of SESN2 partially rescues the development of emphysema in mouse models by activation of PDGFRβ signaling or TGFβ signaling [122–124]. These results suggest that COPD patients might benefit from antagonists of Sesns function [122].

Airway remodeling is an important factor associated with the severity of lung function reduction in COPD. Zhang et al. found that the serum SESN2 level is positively related to airway remodeling [18]. This suggests that SESN2 may be a novel biomarker for prognosis evaluation of COPD patients.

A recent clinical study demonstrated a relationship between SESN2 and asthma [109]. Both during and after asthma exacerbation, the SESN2 level increased [121, 125]. The imbalance between oxidative stress and antioxidant activity in severe asthma patients may explain the change of Sesn level [125].

OSA is characterized by repeated apnea during sleep and intermittent hypoxia, which can lead to serious complications, including coronary heart disease, type 2 diabetes, hypertension, cerebrovascular accident, and stroke [119, 120]. Intermittent hypoxia and the following oxidative stress may cause these complications, which led to research on stress-inducible proteins such as Sesns. Plasma and urinary SESN2 levels were found to increase in OSA patients and to be associated with the severity of OSA, implying that SESN2 can be an important marker of the severity of OSA and the effect of treatment [119, 120, 126].

### Urinary system diseases

Sesns are assumed to play a critical protective role in the kidneys with its functions of mediating stressors such as oxidative stress, ERs, mitochondrial dysfunction, and autophagy, as well as attenuating inflammation and fibrosis [127]. Indeed, studies have shown that SESN2 is involved in acute kidney injury (AKI), glomerular parietal epithelial cells (PECs) injury, glomerular mesangial cell (MC) damage, and diabetic kidney disease (DKD) [127].

SESN2 is upregulated in proximal tubular cells during I/R-induced AKI in vivo, while overexpression of SESN2 induced autophagy in renal tubular cells [87]. Decreased expression of SESN2 in the renal proximal tubules causes ROS overproduction, high renal vascular blood pressure, and renal hypofunction [43]. In addition to AKI, SESN2 has been reported to confer protection in PECs, MCs, and DKD [43]. The mechanism may relate to Sesns’ anti-apoptosis effects, regulation of mTOR, activation of AMPK/Nox4, etc. [128, 129].

DKD, a common diabetic complication that causes end-stage renal disease, is the major cause of chronic kidney disease worldwide [127]. In a human proximal tubule cell line (HK-2) model, over-expression of SESN2 repressed DKD-induced epithelial-mesenchymal transition and ER stress, demonstrating the therapeutic function of SESN2 in DKD [130]. Additionally, SESN2 is reported to improve mitochondrial dysfunction in podocytes under high glucose conditions [131].

Gout is a common type of arthritis caused by elevated serum uric acid (SUA) levels. *SESN2* is identified to be one of the genes that potentially influence SUA, providing insights into the functions of Sesns in the pathogenesis, treatment, and prevention of hyperuricemia/gout [132].

### Immune system related diseases

Sesns are expressed in multiple immune cells such as macrophages, monocytes, NK cells, and T lymphocytes [85, 95, 97, 133]. Sesns suppress the inflammatory response, inhibit T cell immunity, and support macrophage survival [85, 92, 134]. The expression of Sesns might impact the function of immune cells by activating AMPK, suppressing mTORC1 signaling, inhibiting the JNK pathway, or inhibiting the NLRP3 inflammasome from consistent activation [62].

Macrophages are the first line of immune cells that can recognize and eliminate endotoxin [135]. A previous study showed that NO and hypoxia up-regulate SESN2 in macrophages [91]. Lipopolysaccharide (LPS), a representative Toll-like receptor 4 ligand, significantly increases SESN2 expression in macrophages [136]. The Toll-like receptor-mediated induction of SESN2 is dependent on the Nrf2-ARE pathway, AP-1, and the suppression of ubiquitin-mediated degradation of SESN2 and may protect cells against endotoxin toxicity [136].

NK-92 cells are widely used for immunotherapy in cancer due to their high tumoral potency [97]. SESN2 and SESN3 expression levels of NK-92 cells were found to be much higher in ovarian cancer mouse samples, indicating that the tumor microenvironment increased the expression of Sesns [97]. Moreover, overexpression of SESN2 and SESN3 impaired the tumoricidal effect of NK-92 cells, suggesting that downregulating Sesns expression may benefit NK-92 cell-based cancer therapy.

### Musculoskeletal system diseases

**Fig. 2:**
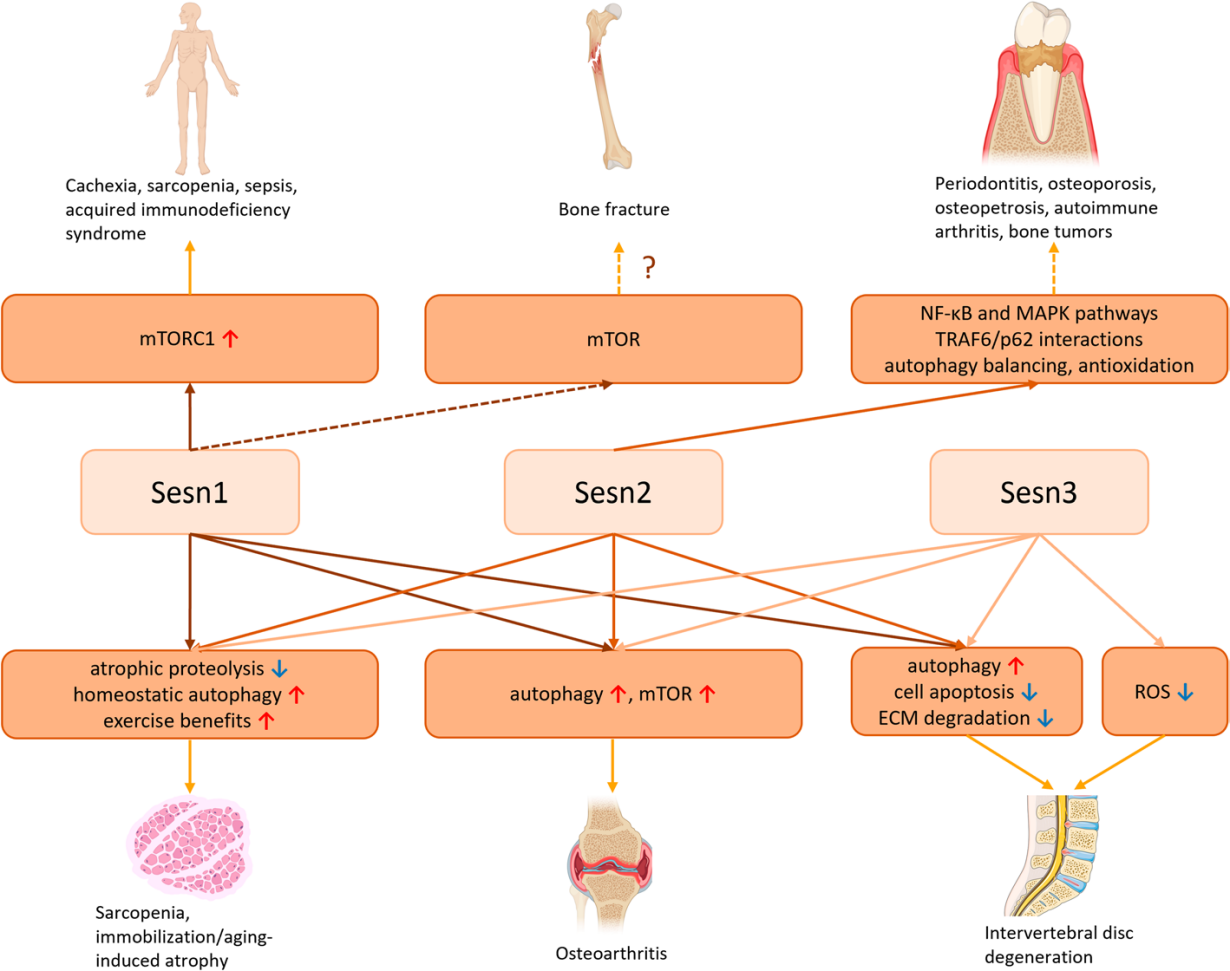
Functions of Sesns in musculoskeletal system diseases. Sesns play important protective roles in multiple musculoskeletal system diseases, such as diseases related to muscle atrophy, bone and skeletal disorders related to osteoclasts, osteoarthritis, intervertebral disc degeneration, etc. The role of Sesns in bone fracture is unproved. The red up arrows represent activation, while the blue down arrows represent inhibition in this figure. Sesns, sestrins; mTOR, mammalian target of rapamycin; mTORC1, mechanistic target of rapamycin complex 1; MAPK, mitogen-activated protein kinase; TRAF6, TNF receptor associated factor 6; NK-κB, nuclear factor kappa-light-chain-enhancer of activated B cells; ECM, extracellular matrix

The musculoskeletal system includes the skeletal system, which comprises bones and cartilages, and the muscular system, which comprises all the body muscles. Recent studies have demonstrated the function of Sesns in musculoskeletal system diseases, such as IDD, osteoarthritis (OA), and sarcopenia. (Fig. 2).

IDD is the primary cause of low back pain and the main factor of functional disability, which significantly affects the quality of life among the elderly population and in some young people [137]. The degeneration of intervertebral disc tissue starts before the degeneration of other musculoskeletal tissues and is often asymptomatic [137]. Although the pathophysiology of IDD is not yet well understood, it is associated with cell senescence, excessive apoptosis, impaired autophagy, pro-inflammatory cytokine storm, and degradation of the extracellular matrix (ECM) [138–141]. As suppressors of cell aging and promotors of autophagy, Sesns are thought to be involved in the IDD process [70, 141, 142]. Tu et al. demonstrated that the expression of SESN1, 2, and 3 markedly decreased in degraded human nucleus pulposus cells but increased after stimulation by an ER stress inducer [70]. Also, knockdown of SESN2 in stress-induced nucleus pulposus cells notably increased cell apoptosis and ECM degradation, while SESN2 overexpression repressed IDD by enhancing autophagy [70]. Consequently, inhibition of Sesns may be related to an essential cellular dysfunction mechanism in IDD. A recent study showed that FoxO, a family of transcription factors that regulate tissue homeostasis and longevity, was reduced in the degraded lumbar intervertebral disc [16]. As a key FoxO downstream target, SESN3 is also decreased in aged lumbar discs, which may compromise the ability of intervertebral disc cells to neutralize ROS [16].

OA is the most prevalent joint disease that affects all synovial joints (hand, hip, knee, and spine) [143]. Risk factors for OA include genetic tendency, aging, metabolic disorders, obesity, previous injury, negative lifestyle, and female gender [144]. OA is mainly characterized by progressive degradation of the articular cartilage and accompanying secondary episodic synovitis and bone remodeling [143]. Several studies show that Sesns play a protective role in cellular homeostasis in OA cartilage [20, 145]. PCR and immunohistochemistry results show that SESN1, SESN2 and SESn3 expression is suppressed in OA-affected cartilage [20]. In siRNA-mediated all Sesns knockdown and SESN2 overexpression experiments, Sesn supports chondrocyte survival under stress conditions by activating autophagy through mTOR signaling [20].

Sarcopenia is an age-related disease of skeletal muscle mass loss. The pathologies are connected with many factors, including inactivity, malnutrition, degeneration of neuromuscular junction, and skeletal muscle cell senescence [146, 147]. Muscle mass is related to survival under several pathological conditions, including sepsis, acquired immunodeficiency syndrome, sarcopenia, and cachexia [148]. Reduced activity, decreased appetite, and nutrient consumption may contribute to the muscle loss. Studies from *Drosophila* and mouse models found that knockout of Sesns resulted in muscle degeneration, providing a connection between Sesns and muscle growth [40]. Segalés and colleagues identified SESN1 and SESN2 as protectors of the muscle against aging-induced atrophy, probably through the inhibition of atrophic proteolysis and activation of homeostatic autophagy [147]. Interestingly, recent studies have shown that Sesns could maintain the homeostasis of muscle stem cells against aging and metabolic insults by inhibiting mTORC1 and maintaining the quiescent state [149].

Regular exercise is an effective intervention to slow down the progression of sarcopenia and increase muscle mass [150, 151]. Sesns have recently been reported to be strongly associated with exercise benefits [110, 152–154]. Kim et al. showed that Sesns are molecular transducers of the beneficial effects of exercise, including enhanced endurance and improved insulin signaling [153]. Sesns expression decreases during inactivity [147]. Loss of Sesns inhibits exercise benefits, while overexpression of them reverses the immobilization/aging-related atrophy [151, 153]. Sestrin proteins are differentially regulated in different training models [155]. Aerobic exercise increases SESN2 protein expression [156], while acute resistance exercises transiently regulate SESN2 [155]. Liu et al. found that long-term endurance exercise raised the protein expression of SESN2 and SESN3, and the basic level of muscle autophagy [110]. Nevertheless, in a recent study, the protein or mRNA expression level of SESN2 and SESN3 or the basal phosphorylation state of SESN2 was not modified after a 12-week long-term resistance training program, whereas the protein expression of SESN1 was induced in human skeletal muscle [155].

Apart from endurance exercise, dietary supplementation of essential amino acids can also lessen the loss of muscle mass [157]. Among all the essential amino acids, leucine is of critical importance since it stimulates skeletal muscle protein synthesis to the same degree as that of a complete mixture of amino acids [158]. Activation of mTORC1 is essential for muscle protein synthesis (MPS) after protein feeding [159–161]. Sesns play an important role in mTORC1 regulation and SESN2 has been considered as a leucine sensor [10, 162]. The expression of Sesn isoforms differs among various tissues. In the skeletal muscle, SESN1 was more abundant than SESN2 and SESN3. Studies have implicated that oral administration of leucine to fasted rats promotes the dissociation of SESN1 from GATOR2 rather than SESN2 or SESN3, indicating that SESN1 regulates leucine-induced activation of mTORC1 in skeletal muscle [162, 163].

Fractures, mostly caused by injury, are significant public health burdens. The physiological process of fracture healing involves a series of well-organized events, including the recruitment of regulatory factors and cell types [164]. Recent studies have suggested that mTOR signaling may be involved in regulating cartilage development and pre-osteoblast differentiation [165]. As negative regulators of mTOR signaling, Sesns are thought to be involved in fracture healing. However, a recent study showed that serum SESN1 levels in bone fracture patients did not differ from those in healthy people, while mTOR levels increased significantly [166]. A limitation of this study is that mTOR and SESN1 levels were only measured on the first day after a fracture. Further clinical studies are needed to explore the potential role of mTOR signaling in the fracture healing process.

A large variety of bone diseases, such as periodontitis, osteoporosis, osteopetrosis, autoimmune arthritis, and bone tumors, are related to the homeostatic equilibrium of bone formation and bone resorption [167–170]. A recent study in mice showed that SESN2 influences bone remodeling and osteoclast differentiation by NFATc1 activation and TRAF6/p62 interaction [171]. Abnormal formation of osteoclasts is vital in various bone and skeletal disorders, so the exact role of Sesns in the maintenance of bone homeostasis and treatment of bone remodeling related bone diseases deserves further investigation.

### Cancer

Cancer is strongly associated with oxidative stress, gene mutation, and metabolic dysregulation. Unlike other cells, cancer cells favor conditions of oxidative stress. It has been reported that mTOR hyperactivation could lead to tumorigenesis and tumor progression [2]. Therefore, as ROS and mTOR inhibitors, Sesns may confer tumor suppressor activity, and be employed in the diagnosis and treatment of multiple cancers [2, 3]. Accumulating evidence demonstrates that most forms of cancers are accompanied by remarkable change of Sesn expression. Sesns can suppress cell growth and proliferation in cancers such as colorectal cancer, lung carcinoma, and endometrial cancer [33, 52, 172–174].

To survive in a hypoxic tumor microenvironment, most cancer cells induce expression of HIF-1α [2, 175]. A study by Seo et al. demonstrated that overexpression of SESN2 suppressed the accumulation of HIF-1α, hence preventing the metastasis of colorectal cancer [6]. Clinical evidence from patients with colon cancer showed that the expression of SESN2 was downregulated and SESN2 levels were negatively correlated with chemotherapy resistance, which further supports the view that SESN2 can serve as a tumor-suppressive protein and a feasible prognostic marker in various cancers such as NSCLC and colon cancer [2]. However, a recent study by Shin et al. showed that SESN2 levels increased in endometrial cancer cells [174]. The study also proved that knockdown of SESN2 could promote cancer cell growth, migration and ROS accumulation via the mTORC1 pathway, indicating the anti-cancer potential of SESN2 and mTORC1 pathway inhibitors in endometrial cancer. Ding et al. reported that SESN2 can inhibit the development of lung adenocarcinoma by regulating X-linked inhibitor of apoptosis protein and inducing cell death through the activation of death receptors [52].

Sesns are also vital in maintaining the viability of cancers under specific conditions [53, 83, 176, 177]. For instance, SESN2 supported the survival of melanoma cells and SCC cells after ultraviolet B radiation and chemotherapeutics [53], and hepatocellular carcinoma cells under glucose limitation [37]. These findings indicate that Sesns may promote tumorigenesis and chemoresistance of cancer cells [53, 97]. In addition, the activation of SESN2 in tumor cells can induce an autophagic response, thus facilitating the growth of tumor cells under limited oxygen and nutrient conditions [83]. Further investigations are needed to illustrate the advantageous or disadvantageous roles of Sesns in cancers and to determine their potential applications in radiotherapy or chemotherapy [3].

## Future directions: Sesns as biomarkers and therapeutic targets of diseases

**Fig. 3:**
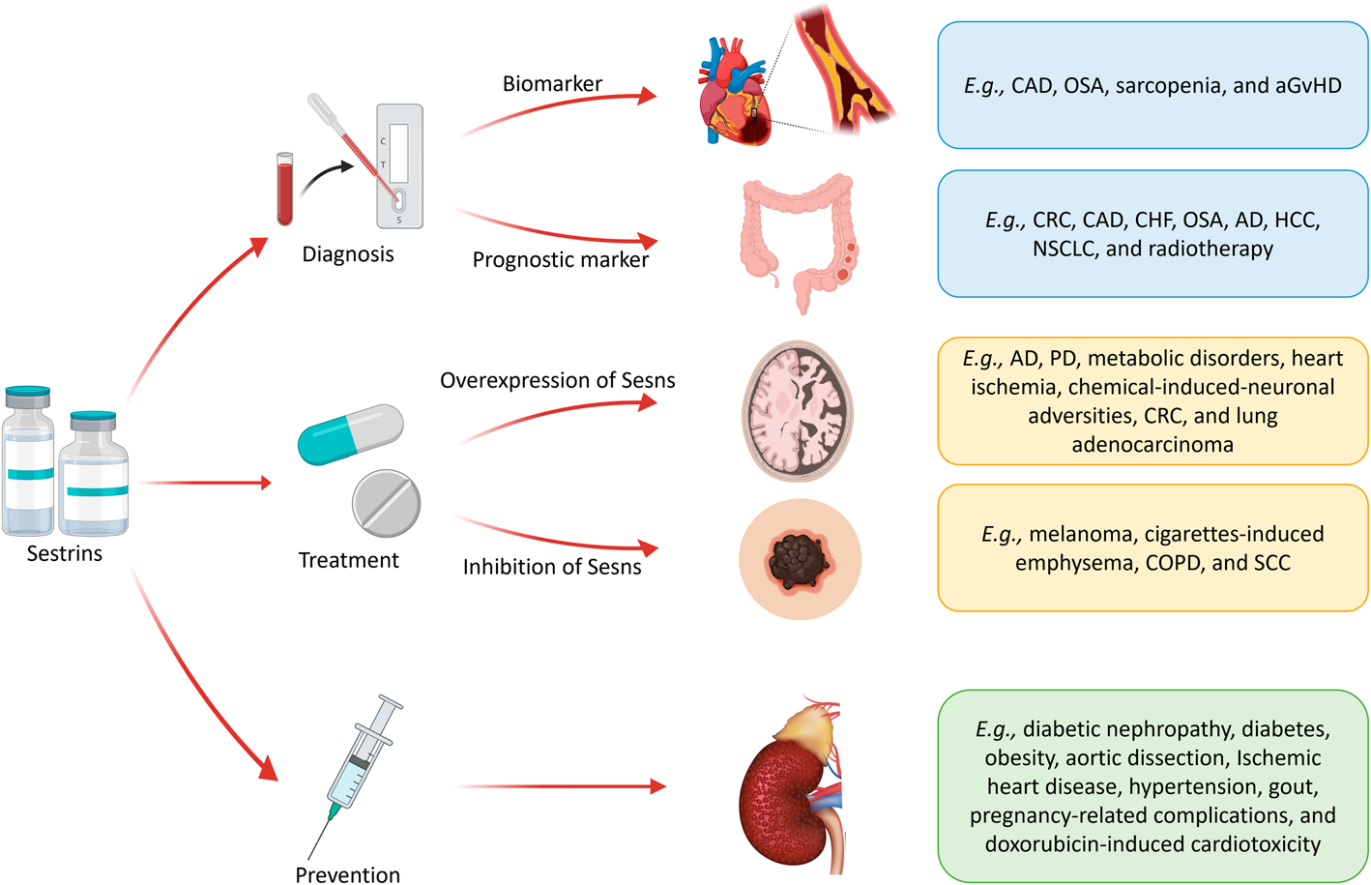
Small-molecule inducers or activators targeting Sesns may be used in diagnosis, treatment, and prevention of human diseases. Sesn, sestrin; CAD, coronary heart disease; OSA, obstructive sleep apnea; aGvHD, acute graft-versus host disease; CRC, colorectal cancer; CHF, chronic heart failure; AD, Alzheimer's disease; HCC, hepatocellular carcinoma; NSCLC, non-small-cell lung cancer; PD, Parkinson’s disease; COPD, chronic obstructive pulmonary disease; SCC, squamous cell carcinoma

Given these multiple effects, members of the Sesns protein family are potential biomarkers and treatment targets in aging-related diseases, metabolic diseases, bone diseases, and various other diseases [2]. Here, we list the potential functions of Sesns and their regulators in modern medicine (Fig. 3).

### Sesns for disease diagnosis

Sesns levels can reflect the status of many diseases, which could be used as valuable biomarkers for diagnosis. For instance, the SESN2 level was suggested to be an early biomarker of atherogenesis, CAD, OSA and sarcopenia [17, 94, 103]. The sensitivity and specificity of SESN2 for OSA diagnosis were respectively 61.90% and 90.70%, which are clinically valuable levels [126]. What is more, SESN3 as a colon-specific marker might improve the detection of acute graft-versus-host disease (aGvHD) [178].

Sesns levels could also be prognostic markers for predicting the treatment outcome. Plasma or urinary SESN2 levels were reported to reflect the severity of CAD, CHF, coronary stenosis, OSA and COPD [102, 103, 119]. SESN2 levels might be positive prognostic markers in HCC [179], NSCLC [172], and neurodegenerative diseases such as AD [2]. They could be used to predict poor outcomes for patients with CHF [102] or colorectal cancer [173, 180]. Apart from that, SESN1 is associated with individual radiosensitivity, which could be utilized for predicting the toxicity of radiotherapy for cancers [181].

### Sesns for disease treatment

Studies have shown that Sesns may be novel therapeutic drug targets. Small-molecule Sesns mimetics, inducers or activators might reverse pathologic conditions or diseases, such as heart ischemia**,** metabolic disorders, AD, PD, chemical-induced-neuronal adversities, colorectal cancer, and lung adenocarcinoma [2, 52, 182, 183]. Sesns antagonists and inhibitors or siRNA drugs that could knock down Sesns might confer therapeutic benefits for diseases such as CS-induced emphysema, COPD [123], SCC, hepatocellular carcinoma, ovarian cancer, and melanoma [53]. In addition to drugs with direct effects, Sesns could also be used to design adjuvant therapies. Based on a recent study, SESN2 might be an effective preclinical target for colorectal cancer in chemotherapy combined with nutritional supplements [184]. Pro-oxidant drugs that promote the protective effect of SESN1 in cancer cells might provide new therapeutic opportunities for cancer patients bearing the mutant TP53 gene [185]. Drugs that induce Sesns-mediated autophagy and inhibit growth of cancer cells could be a novel weapon against cancers such as human bladder cancer [186]. More importantly, Sesns might be a good target to overcome resistance of anticancer drugs, which is one of the main obstacles that influence cancer treatment [187].

In recent years, genetically modified cell sheets using virus-based or non-viral gene transfection have shown great potential in personalized and precision medicine [188]. Sesns-modified cell sheets might act as a promising preventive or therapeutic drug against aging-related diseases such as sarcopenia, cancers, AD, PD, and CAD. However, the diagnostic and therapeutic benefits of Sesns can only be obtained when its upstream and downstream pathways that underlie their biological effects are well understood.

### Sesns for disease prevention

Sesns have also been reported to prevent diseases such as aortic dissection, ischemic heart disease, diabetes, insulin resistance, obesity, and hyperuricemia/gout [107, 108, 132, 189, 190]. Patients with hypertension showed elevated circulating Sesns levels, which provides a clue for preventing clinical hypertension [190]. SESN2 can also prevent pregnancy-related complications given that it was found to correct impaired trophoblast invasion, ER stress and inflammation caused by palmitate [191]. Patients with diabetic nephropathy showed decreased serum SESN2 levels; thus, measurement of SESN2 levels may be an effective approach for early detection and prevention of diabetic nephropathy [192]. Doxorubicin, a highly efficient chemotherapeutic medicine, is associated with high cardiotoxicity. A study showed that Sesns counteracted the detrimental effects of doxorubicin on cardiomyocytes, without causing cardiotoxicity [193]. It is therefore likely to be an important prevention and treatment agent against doxorubicin-induced cardiotoxicity.

Dietary restriction can increase longevity and improve health in diverse species [59]. Restriction of specific essential amino acids plays a key role, but the molecular and cellular mechanisms are still elusive [60]. Recent studies show that Sesns may be the link between dietary amino acids, intestinal stem cell function, gut health, and lifespan by regulating the mTOR pathway and autophagy, which could provide another clue for human disease prevention [61].

## Conclusion

Sesns, stress-inducible metabolic proteins that repress ROS and provide cytoprotection against various noxious stimuli, have aroused great interest recently. Considering their pleiotropic functions including cellular stress elimination, AMPK promotion/mTORC1 repression, autophagy induction, pro-survival effects on normal cells as well as anti-proliferative effects on cancerous cells, Sesns are key regulators of cell metabolism and contribute to cell homeostasis in physiological and pathological conditions. Owing to their antioxidant function, Sesns protect tissues in neurodegenerative disorders such as PD and AD. As an activator of AMPK and an inhibitor of mTORC1, Sesns help animals fight against various metabolic disorders, such as diabetes, obesity, cancer, atherosclerosis, and cardiac hypertrophy. Therefore, Sesns can serve as prognostic indicators and potential therapeutic targets in many disorders. Despite the protective roles of Sesns, whether uncontrolled activation of them would result in negative impacts should also be ascertained. Future studies based on transgenic animal models with silencing of Sesns should be developed to test small molecule Sesns mimetics or agonists in various diseases. Personalized medicine targeting Sesns is promisingly envisioned to develop from bench to bedside in the next generation.

## Data Availability

Not applicable.

## Abbreviations

AD: Alzheimer’s disease
AKI: Acute kidney injury
ALS: Amyotrophic lateral sclerosis
AMI: Acute myocardial infarction
AMPK: AMP-activated protein kinase
AP-1: Activator protein 1
ARDS: Acute respiratory distress syndrome
ARE: Antioxidant responsive element
AS: Atherosclerosis
ATF4: Activating transcription factor 4
ATF6: Activating transcription factor 6
aGvHD: Acute graft-versus-host disease
C/EBPβ: CCAAT/enhancer-binding protein beta
CAD: Coronary heart disease
CHF: Chronic heart failure
CHOP: CCAAT/enhancer-binding protein (C/EBP) homologous protein
COPD: Chronic obstructive pulmonary disease
CVDs: Cardiovascular diseases
DKD: Diabetic kidney disease
ECM: Extracellular matrix
ER: Endoplasmic reticulum
Erk: Extracellular signal-regulated kinase
ERs: Estrogen receptors
FoxO: Forkhead box protein O
GADD: Growth arrest and DNA damage-inducible genes
GATOR: GTPase-activating protein (GAP) activity toward Rags
HCC: Hepatocellular carcinoma
HD: Huntington’s disease
HIE: Hypoxic-ischemic encephalopathy
HIF1: Hypoxia-inducible factor 1
I/R: Myocardial ischemia and reperfusion
IDD: Intervertebral disc degeneration
IgE: Immunoglobulin E
IL-2: Interleukin 2
IRE1: Inositol-requiring enzyme 1
JNK: C-Jun N-terminal kinase
Keap1: Kelch-like ECH-associated protein 1
LPS: Lipopolysaccharide
MAPK: Mitogen-activated protein kinase
MCs: Glomerular mesangial cells
MPS: Muscle protein synthesis
mTOR: Mammalian target of rapamycin
mTORC1: Mechanistic target of rapamycin complex 1
mTORC2: Mechanistic target of rapamycin complex 2
NAFLD: Nonalcoholic fatty liver disease
NFATc1: Nuclear factor of activated T-cells c1
NK: Natural killer
NK-κB: Nuclear factor kappa-light-chain-enhancer of activated B cells
NLRP3: Nod-like receptor family pyrin domain containing 3
NMDA: N-methyl-d-aspartate
Nox4: NADPH oxidase 4
Nrf2: Nuclear factor erythroid 2-related factor 2
OSA: Obstructive sleep apnea
PCR: Polymerase chain reaction
PD: Parkinson’s disease
PDGFRβ: Platelet-derived growth factor receptor beta
PECs: Glomerular parietal epithelial cells
PERK: Protein kinase RNA-like endoplasmic reticulum kinase
PGC-1α: Peroxisome proliferator-activated receptor-gamma coactivator alpha
PHD: Prolyl hydroxylase
PI3K: Phosphoinositide 3-kinase
PKB: Protein kinase B, also known as Akt
*RAGs*: Recombination-activating genes
RBX1: Ring-box 1
RNS: Reactive nitrogen species
ROS: Reactive oxygen species
S6K: Ribosomal protein S6 kinase
SCC: Squamous cell carcinoma
Sesn: Sestrin
siRNA: Small interfering RNA
SQSTM1: Sequestosome 1
SUA: Elevated serum uric acid
TGFβ: Transforming growth factor β
TLR4: Toll like receptor 4
TRAF6: TNF receptor associated factor 6
ULK1: Unc-51-like kinase 1
UPR: Unfolded protein response
VEGF: Vascular endothelial growth factor
XBP1: X-box binding protein 1
XIAP: X-linked inhibitor of apoptosis protein
